# Pluripotent stem cells for the study of early human embryology

**DOI:** 10.1111/dgd.12715

**Published:** 2021-03-22

**Authors:** Katsunori Semi, Yasuhiro Takashima

**Affiliations:** ^1^ Center for iPS Cell Research and Application Kyoto University Kyoto Japan

**Keywords:** early embryonic development, naïve pluripotent stem cells, pluripotent stem cells, primed pluripotent stem cells, synthetic embryo

## Abstract

Forty years have passed since the first pluripotent stem cells (PSCs), mouse embryonic stem cells (ESCs), were established. Since then, several PSCs have been reported, including human ESCs in 1998, mouse epiblast stem cells (EpiSCs) in 2007, induced PSCs (iPSCs) in 2006 and 2007, and naïve human PSCs in 2014. Naïve PSCs are thought to correspond to pre‐implantation epiblast cells, whereas conventional (or primed) human PSCs correspond to post‐implantation epiblast cells. Thus, naïve and primed PSCs are classified by their developmental stages and have stage‐specific characteristics, despite sharing the common feature of pluripotency. In this review, we discuss the current status of PSCs and their use to model human peri‐implantation development.

## Graphical abstract

Naïve and primed pluripotent stem cells correspond to pre‐implantation and post‐implantation epiblast, respectively. Both show pluripotency but different characteristics and function

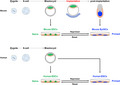

## 1 INTRODUCTION

The establishments of mouse and human embryonic stem cells (ESCs) were separated by more than 15 years (Evans & Kaufman, 1981; Martin, 1981; Thomson et al., 1998). This lag was primarily due to the fact that the culture condition for mouse ESCs did not work for human ESCs. Specifically, leukemia inhibitory factor (LIF), a key factor for the self‐renewal of mouse ESCs (Smith et al., 1988; Williams et al., 1988), does not provide a sufficient signal to maintain human ESCs. Instead, FGF and TGFβ/ACTIVIN signaling were found essential to maintain human pluripotency (Ludwig et al., 2006; Vallier et al., 2005). Since the founding of ESCs, other major pluripotent stem cells (PSCs) reported included induced pluripotent stem (iPS) cells (Takahashi & Yamanaka, 2006) and mouse epiblast stem cells (EpiSCs) (Brons et al., 2007; Tesar et al., 2007). Notably, like human PSCs, mouse EpiSCs depend on FGF and ACTIVIN. They also correspond to post‐implantation epiblast. Other similarities between mouse EpiSCs and human PSCs include a flat shape morphology, metabolism, signaling pathways, and epigenetic patterns (Table 1). They also share the same pluripotency, primed pluripotency, whereas mouse ESCs exhibit naïve pluripotency. Naïve human PSCs were finally reported in 2014 (Takashima et al., 2014; Theunissen et al., 2014). Much of the knowledge gained from mouse ESCs was used to establish culture systems that maintain naïve human PSCs. In particular, the establishment of two‐inhibitor (2i) medium (Ying et al., 2008), which contains a MEK inhibitor, PD0325901 (PD03), and a GSK3 inhibitor, CHIR99021 (CHIR), enabled the establishment of ESCs from non‐obese diabetic (NOD) mice (Nichols et al., 2009) and other rodents, such as rats (Buehr et al., 2008; Li et al., 2008), which cannot be established using the traditional serum + LIF medium. Notably, 2i medium was fundamental for establishing the original naïve human PSCs. Since naïve human PSCs have the characteristics of the pre‐implantation epiblast (Nakamura et al., 2016; Stirparo et al., 2018; Takashima et al., 2014; Theunissen et al., 2014), they are viewed as models of human peri‐implantation development in vitro.

**TABLE 1:** Comparison of naïve and primed pluripotent stem cells.

	Mouse ESCs	Mouse EpiSCs	Naïve human PSCs	Primed human PSCs
Pluripotent state	Naïve	Primed	Naïve	Primed
MEK–ERK dependence	No	Yes	No	Yes
Dependence on FGF2 signaling	No	Yes	No	Yes
Dependence on JAK/STAT signaling	Yes	No	Not confirmed^a^	No
Colony morphology	Dome shape	Flat	Dome shape	Flat
Metabolism	OxPhos, glycolytic	Glycolytic	OxPhos, glycolytic	Glycolytic
Global DNA hypomethylation	Yes	No	Yes	No
Differentiation to three germ layers	Yes	Yes	Yes^b^	Yes
Differentiation to germ lineage cells	Yes	No	Not tested	Yes
X chromosome activation	Yes	No	Yes	No
Capacity of contribution to chimeras	High	Low	Not tested	Not tested
Capacity of contribution to germline chimeras	Yes	No	Not tested	Not tested

^a^ JAK/STAT signaling is activated in naïve human PSCs, but it has not been confirmed whether this signaling is essential.

^b^ Naïve human PSCs cannot directly differentiate into three germ layers but differentiate into three germ layers via primed state human PSCs.

Ideally, human embryos would be used to study human peri‐implantation development, but access to them is limited due to ethical reasons. Furthermore, analysis of embryonic development at the post‐implantation stage is forbidden, as this stage occurs in utero. Overall, very few reports offer histological data (Hertig & Rock, 1949; O’Rahilly & Muller, 1987); instead, mouse models are commonly used. However, single‐cell RNA sequencing (scRNA‐seq) data have shown that the pre‐implantation embryos of humans and mice exhibit species‐specific gene expression patterns (Blakeley et al., 2015; Boroviak et al., 2018). To understand the post‐implantation stages of development in humans, several technological approaches have been attempted. In this review, we examine the methods available for analyzing both the pre‐ and post‐implantation stages of development in humans. In particular, we describe long‐term ex vivo embryo cultures for mice, non‐human primates, and humans and the construction of ESC‐derived spheroids using aggregates of PSCs with or without extraembryonic lineage cells. We also discuss naïve human PSCs and their potential to analyze human embryonic development.

## 2 ESTABLISHMENT OF NAÏVE HUMAN PSCs

**FIGURE 1:**
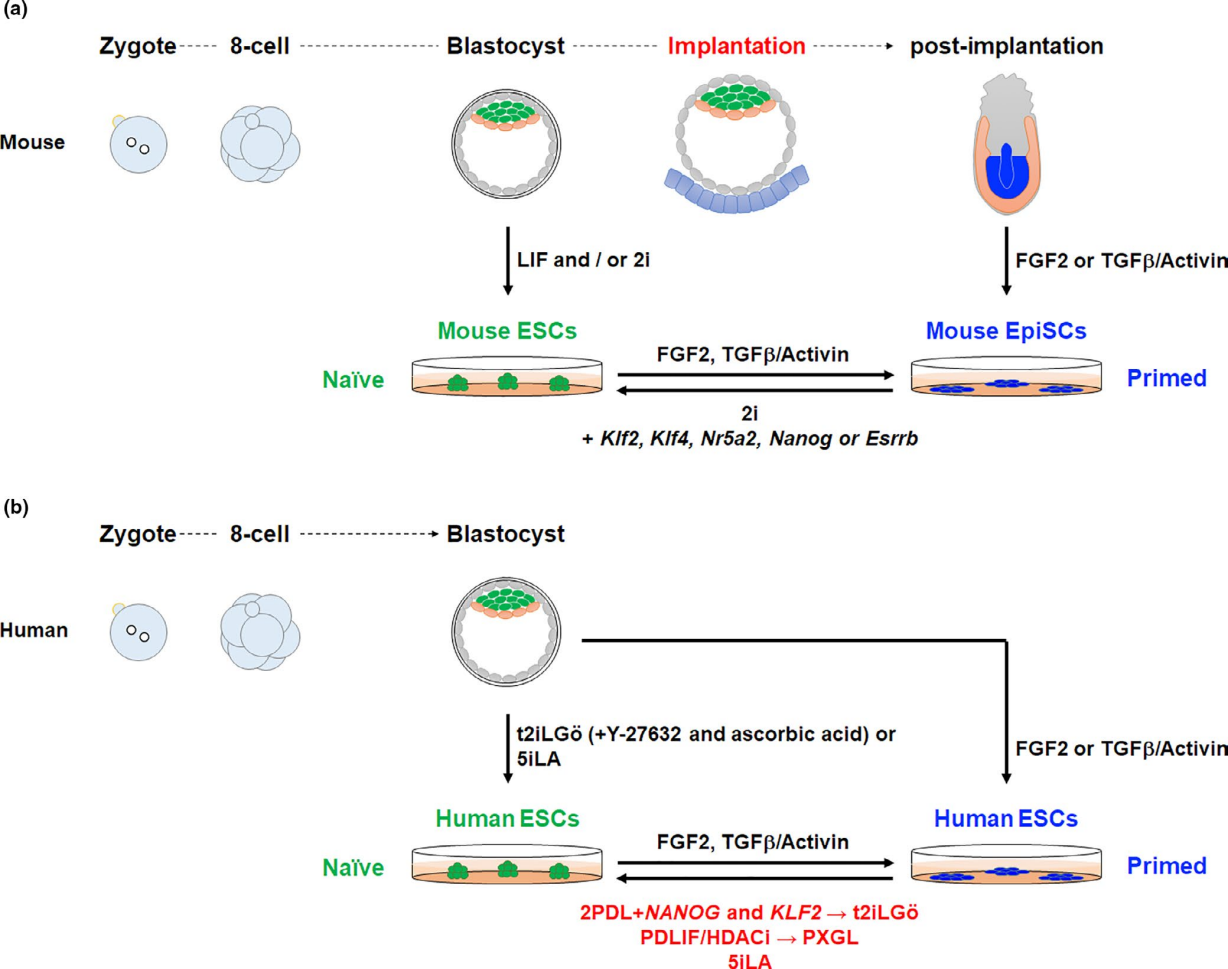
Developmental stages and pluripotent stem cells. (a) Mouse development and pluripotent stem cells (PSCs). After fertilization, the egg starts to cleave and develops into a blastocyst, which contains epiblast, hypoblast (primitive endoderm), and trophectoderm. A blastocyst implants into the uterus. At the egg cylinder stage, the epiblast starts gastrulation to form the three germ layers: endoderm, mesoderm, and ectoderm. Mouse embryonic stem cells (ESCs) are derived from the epiblast of the blastocyst (green cells) under LIF or 2i media. Mouse epiblast stem cells (EpiSCs) are derived from post‐implantation epiblast (blue cells) under FGF2 and TGFβ/Activin. Both are PSCs. Mouse ESCs are called naïve PSCs because they correspond to pre‐implantation epiblast. Mouse EpiSCs are called primed PSCs, because they correspond to post‐implantation epiblast. Mouse ESCs under FGF2 and TGFβ/Activin naturally convert to mouse EpiSCs, while mouse EpiSCs can be reset to naïve PSCs by the overexpression of one of several transgenes (*Klf2*, *Klf4*, *Nr5a2*, *Nanog*, and *Esrrb*). (b) Human development and PSCs. Overall, human fertilized embryos develop similarly to mouse embryos. However, there exist differences in the gene expression profiles, signaling pathways, stage of zygotic genome activation, and morphology. Conventional human ESCs were established under FGF2 and TGFβ/Activin like mouse EpiSCs. Also like mouse EpiSCs, they are primed PSCs, suggesting human ESCs represent post‐implantation epiblast. Naïve human PSCs can be established by two ways. One is to directly establish naïve PSCs from pre‐implantation embryos under human naïve culture medium (t2iLGö or 5iLA). The other is to reset primed PSCs to naïve PSCs under 2PDL (PD03, PD173074, and LIF) with the overexpression of *NANOG* and *KLF2*, PDLIF/HDACi (PD03, LIF, and HDACi) or under 5iLA.

Research has revealed that there are several pluripotent states. The first PSCs reported, mouse ESCs, have naïve pluripotency and resemble pre‐implantation epiblast. On the other hand, mouse EpiSCs have primed pluripotency and represent post‐implantation epiblast. Consistently, these cells were derived from pre‐ and post‐implantation embryonic cells, respectively (Figure 1a) (Nichols & Smith, 2009). Either pluripotency state reflects the potential to differentiate into all types of somatic lineage cells. However, chimeric competency has been detected in mouse ESCs but not EpiSCs (Table 1) (Brons et al., 2007; Guo et al., 2009<). Moreover, mouse EpiSCs do not differentiate into germ lineage cells. Despite being derived from pre‐implantation embryonic cells, human ESCs more resemble mouse EpiSCs than mouse ESCs in terms of colony morphology, signaling dependency for the maintenance of pluripotency, and other properties (Figure 1b and Table 1). For ethical reasons, human embryos are difficult to procure for study of the pre‐ to post‐implantation states. Naïve human PSCs are considered an acceptable substitute.

Mouse EpiSCs can be reset to naïve PSCs by the overexpression of a single transcription factor, such as *Klf2*, *Klf4*, *Nr5a1*, *Nr5a2*, *Nanog*, or *Esrrb* (Figure 1a) (Festuccia et al., 2012; Guo & Smith, 2010; Guo et al., 2009; Hall et al., 2009; Silva et al., 2009), followed by culture in 2i medium (Ying et al., 2008). These findings suggested that primed human PSCs could also be reset to naïve pluripotency. The 2i medium is a serum‐free medium containing PD03 to inhibit FGF signaling and CHIR to activate Wnt signaling and inhibit GSK3 function (Ying et al., 2008). Thus, 2i medium inhibits differentiation cues and induces pluripotent genes. These findings suggest that multiple signaling pathways are involved in maintaining naïve pluripotency.

Over the past decade, various induction protocols for naïve‐like human PSCs have been reported (Chan et al., 2013; Chen et al., ,^2015^, ^2018^; Gafni et al., 2013; Guo et al., 2017; Hanna et al., 2010; Qin et al., 2016; Takashima et al., 2014; Theunissen et al., 2014; Wang et al., 2011; Ware et al., 2014; Zimmerlin et al., 2016). Since chimera formation assays on human PSCs are prohibited, the identification of naïve pluripotency is based on biological features, such as the expression profiles of naïve PSC marker genes, DNA hypomethylation, reactivation of silenced X chromosome, and transposable elements (Boroviak & Nichols, 2017). Comparisons between the global expression profiles of naïve PSCs and scRNA‐seq data of human embryos revealed that naïve human PSCs established by t2iLGö (PD03, CHIR, LIF, and Gö6983) or 5iLA (PD03, IM‐12, LIF, WH‐4–023, Y‐27632, SB590885, and Activin A) media highly resemble pre‐implantation epiblast cells (Table 2) (Huang et al., 2014; Nakamura et al., 2016; Stirparo et al., 2018; Takashima et al., 2014; Theunissen et al., 2014). In addition, t2iLGö and 5iLA media can be used to establish naïve ESCs directly from human pre‐implantation blastocysts (Guo et al., 2016; Theunissen et al., 2014). These two media commonly contain a MEK inhibitor (PD03) and Wnt signal activator (CHIR or IM‐12). Interestingly, the removal of the Wnt signal activator did not cause a significant reduction in *OCT4* distal enhancer activity or *KLF4* expression compared to removal of the MEK inhibitor (Theunissen et al., 2014). In addition, lower concentrations of CHIR in t2iLGö medium induced a more homogenous colony morphology than did lower concentrations in the conventional t2iLGö medium (Guo et al., 2017). Together, these findings indicated that the activation of Wnt signaling may not be required for establishing naïve human PSCs. More recently, PXGL medium, which replaces CHIR in t2iLGö medium with a tankyrase inhibitor, XAV939 (XAV), to suppress Wnt signaling, was found to maintain naïve pluripotency (Table 2) (Bredenkamp et al., 2019; Guo et al., 2017). Indeed, XAV treatment can increase the efficiency of reprogramming to naïve human PSCs (Bredenkamp et al., 2019; Guo et al., 2017). Overall, the above studies show that different media can induce naïve human PSCs from primed PSCs.

**TABLE 2:** Media compositions associated with the induction of naïve hPSCs.

Component	Target	Signaling	2 Step protocol				1 Step protocol
			Takashima et al.		Guo et al.		Theunissen et al.
		Activation/inhibition	2PDL	t2iLGö	PDL/HDACi	PXGL	5iLA
LIF	LIFR/IL6ST	JAK/STAT	**○**	**○**	**○**	**○**	**○**
PD0325901	MAPK	MAPK	**○**	**○**	**○**	**○**	**○**
PD173074	FGFR	MAPK	**○**				
CHIR99021	GSK3β	WNT		**○**			
IM‐12	GSK3β	WNT					**○**
XAV939	Tankyrase	WNT				**○**	
Gö6983	PKC	PKC		**○**		**○**	
WH‐4‐023	SRC	SRC					**○**
Y‐27632	ROCK	RHO/ROCK		**○** ^a^			**○**
SB590885	BRAF	RAF‐ERK					**○**
Activin A	ALK4/ALK7	TGFb/Activin					**○**
Ascorbic acid				**○** ^a^			
VPA	HDAC	HDAC			**○**		
Other conditions		Basal media	N2B27	N2B27	N2B27	N2B27	N2B27
		O_2_ level	5%	5%	5%	5%	5%
		Transgene	*KLF2*, *NANOG*				

Note

Blue font indicates inhibition. Red font indicates activation. Circle indicates the component is contained.

^a^ Y‐27632 and ascorbic acid are added for the derivation from human embryos.

Overexpression of transgenes, such as *NANOG* and *KLF2*, is another way to robustly induce naïve PSCs (Table 2) (Takashima et al., 2014). Overexpressing *KLF4* only in t2iLGö medium can also reset human primed PSCs to naïve PSCs (Liu et al., 2017). Alternatively, chemical‐only induction methods for resetting are available (Table 2). Austin Smith's group reset primed human PSCs by culturing them in PDLIF/HDACi (PD03, LIF, and histone deacetylase inhibitor (HDACi)) for 3 days and then in PXGL (Guo et al., 2017). The addition of HDACi enables the induction of naive human PSCs without any forced expression of transcription factors. However, using the 5iLA medium is the simplest method because primed human PSCs can be reset to naïve PSCs in 5iLA without any medium change (Theunissen et al., 2014). However, karyotypic abnormalities are more likely to occur in 5iLA medium than in t2iLGö or PXGL medium (Guo et al., 2017; Liu et al., 2017). Such karyotypic abnormalities can be reduced by a lower PD03 concentration and alternative MEK inhibitors (Di Stefano et al., 2018).

## 3 MONITORING THE ACQUISITION OF HUMAN NAÏVE PLURIPOTENCY

**FIGURE 2:**
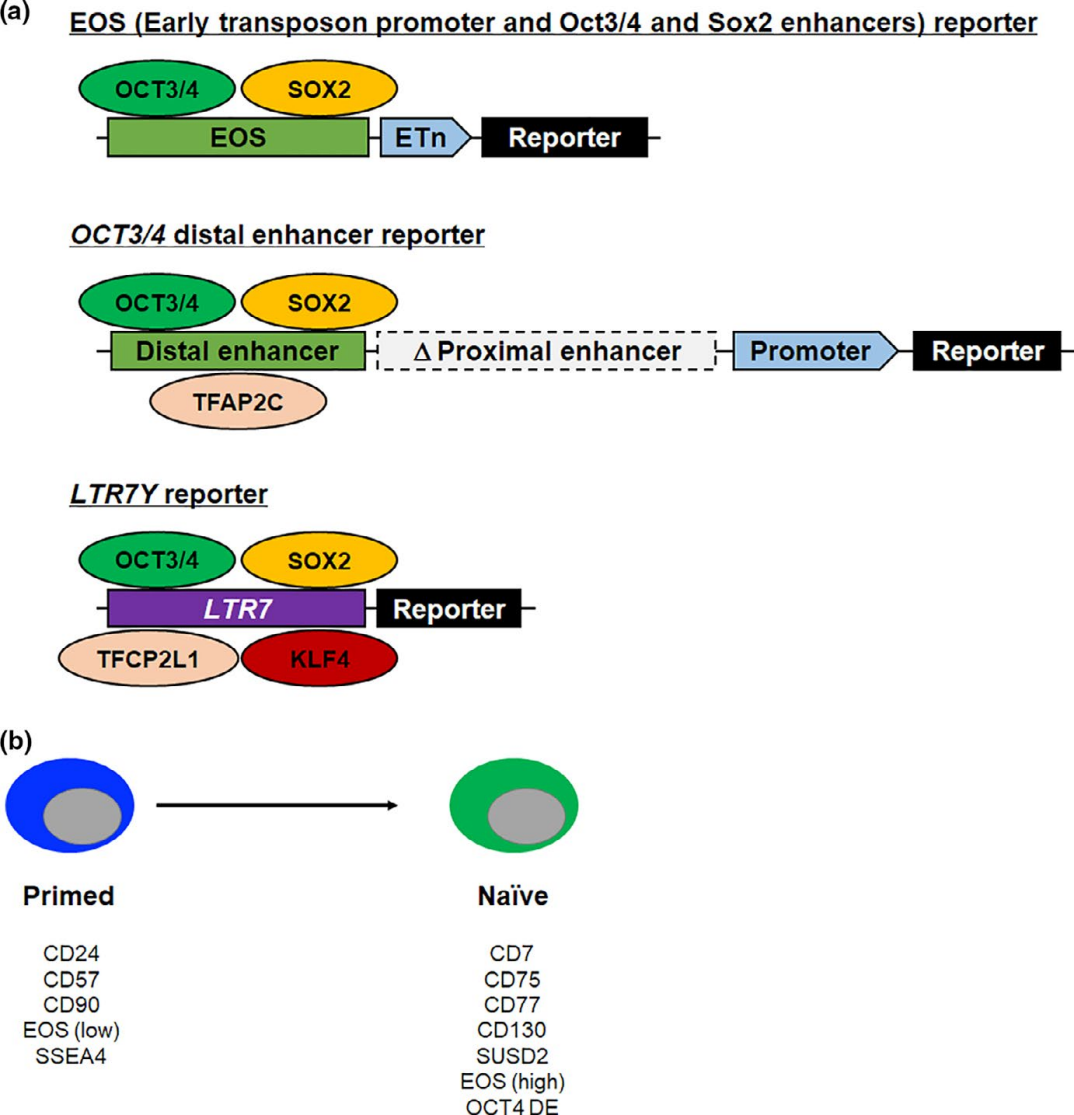
Visualization of naïve PSCs by genetic modification or cell surface markers. (a) Reporter genes for naïve PSCs. Different sets of pluripotency‐related genes (OCT3/4, SOX2, TFAP2C, KLF4, and TFCP2L1) bind to Oct3/4, Sox2 enhancer (EOS), *OCT3/4* distal enhancer, or HERV‐H associated LTR elements. (b) Cell surface markers for naïve and primed PSCs.

One challenge in the resetting process is identifying when and how the pluripotency stage switches from primed to naïve. To visualize naïve PSCs, naïve‐specific reporters and cell surface markers have been used (Figure 2). Han et al. used GOF18‐GFP mouse EpiSCs (Han et al., 2010) that harbor a randomly integrated GFP transgene under the control of an *Oct3/4* distal enhancer sequence (Figure 2a) (Yeom et al., 1996). Since the conversion of the *Oct3/4* regulatory region is conserved not only in mice but also in humans, the *OCT3*/*4* distal enhancer was applied for the screening of chemical compounds of human naïve medium (Theunissen et al., 2014). The early transposon promoter and Oct3/4 (Pou5f1) and Sox2 enhancers (EOS) reporter is another marker of naïve PSCs in both mice and humans (Takashima et al., 2014). The EOS‐reporter plasmid includes a fragment of the *Oct4* distal enhancer, which contains Oct4 and Sox2 binding motifs, and the LTR promoter of an early transposon (ETn) (Figure 2a) (Hotta et al., 2009; Maksakova & Mager, 2005). Reduced DNA methylation and repressive histone modifications (H3K9me3 and H3K27me3) are observed in naïve PSCs and lead to reactivation of the endogenous retrovirus (ERV) element associated with the transcription networks of primed and naïve PSCs (Goke et al., 2015; Lu et al., 2014; Theunissen et al., 2016). However, the EOS plasmid is weakly expressed in primed human PSCs (Hotta et al., 2009). Thus, a highly specific reporter is necessary to monitor putative naïve human PSCs. Some transposable elements are markers of both primed and naïve PSCs, but different types of transposable elements are known to be activated depending on the type of PSCs (Goke et al., 2015; Lu et al., 2014; Theunissen et al., 2016). HERV‐H associated LTR (LTR7, LTR7Y, and LTR7B) elements show developmental stage‐specific expression (Goke et al., 2015). LTR7 elements are associated with the transcription networks of primed PSCs (Lu et al., 2014; Ohnuki et al., 2014). In contrast, activated LTR7Y elements are specifically observed in pre‐implantation blastocysts (Goke et al., 2015). Recently, the promoter activity of LTR7Y element was used as a specific reporter for naïve PSCs (Szczerbinska et al., 2019). The expression of LTR7 elements is induced by the binding of transcription factors that are highly expressed in naïve human PSCs, such as OCT3/4, NANOG, KLF4, and TFCP2L1 (Fort et al., 2014; Lu et al., 2014; Wang et al., 2014). TFAP2C is also known to induce the enhancer activity of *OCT3*/*4* distal enhancer during the primed to naïve transition (Pastor et al., 2018) and is required for *KLF4* expression (Chen et al., 2018) (Figure 2a).

Although genetic reporters allow us to perform cost‐effective and large‐scale screening (Szczerbinska et al., 2019; Theunissen et al., 2014), genetic manipulation is required. In contrast, cell surface markers are useful for isolating live cells by flow cytometry when combined with antibodies and fluorescent dyes. To define stage‐specific cell surface markers during the induction of naïve pluripotency, screening was performed using antibody panels for cell surface proteins, and naïve‐specific cell surface markers (CD7, CD75, CD77, and CD130) and primed‐specific markers (CD24, CD90, CD57, and HLA‐ABC) were defined (Figure 2b) (Collier et al., 2017). Another naïve PSC‐specific cell surface marker, SUSD2, was also reported (Bredenkamp et al., 2019); its expression was confirmed in the epiblast of pre‐implantation human embryos. Both cell surface markers and genetic reporters are useful for elucidating the detailed mechanism of naïve pluripotency in human PSCs.

## 4 DIFFERENTIATION FROM NAÏVE PLURIPOTENCY TO FORMATIVE AND PRIMED PLURIPOTENCY

The transcriptome expression patterns of naïve PSCs correspond to those of the late epiblast of pre‐implantation blastocysts (Boroviak & Nichols, 2014; Nakamura et al., 2016; Stirparo et al., 2018). The most significant difference between naïve and primed PSCs in mice is the potential to differentiate into germ cells both in vitro and in vivo. Naïve mouse PSCs have the ability to differentiate into all types of embryonic lineages and germline cells and can form chimera that also contribute to germ lineage cells. On the other hand, primed PSCs have the ability to differentiate into the three germ layers (ectoderm, endoderm, and mesoderm), but fail to form germline chimeras.

**FIGURE 3:**
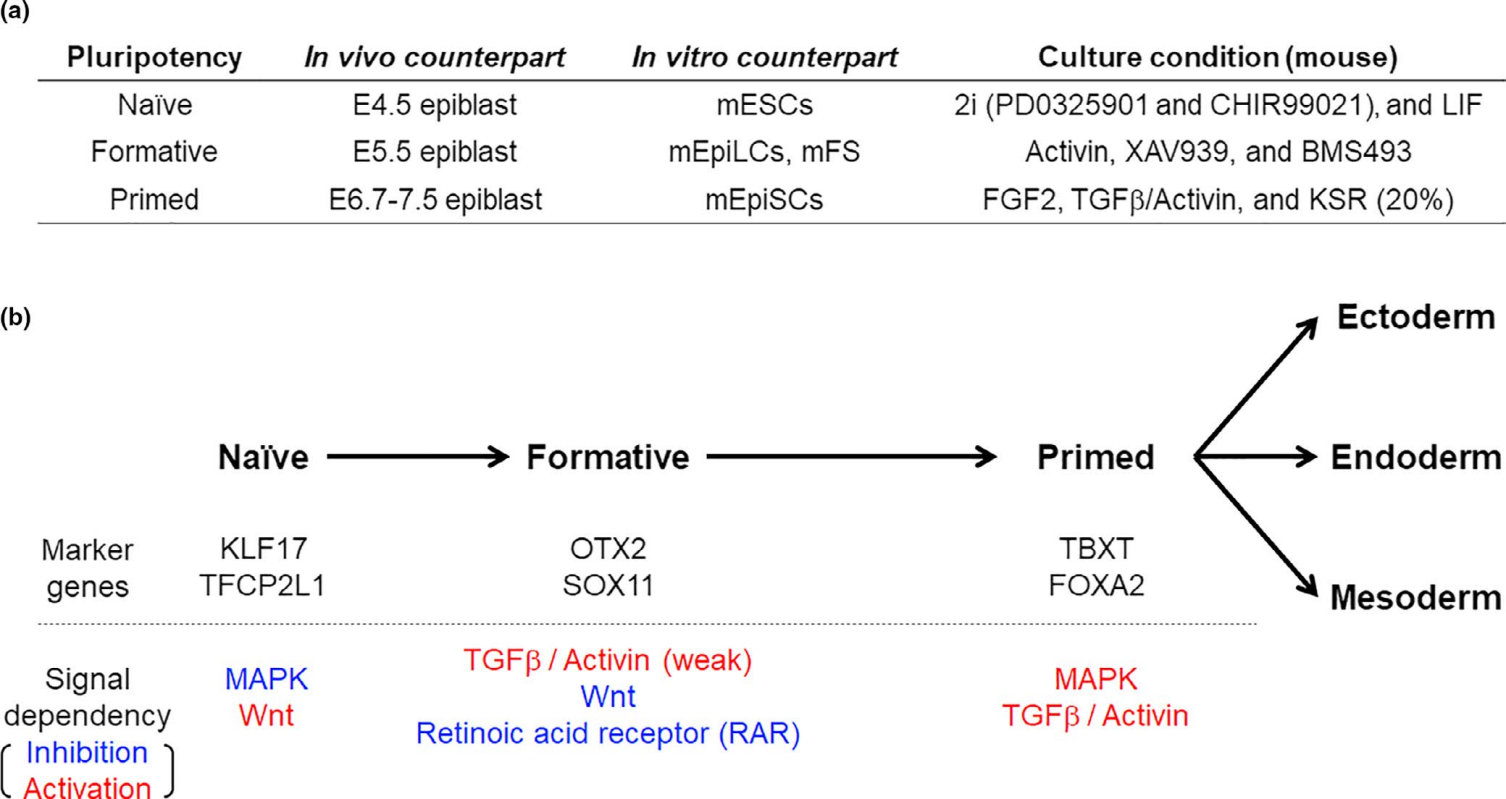
Formative stem cells in mouse and human. (a) Summary of each pluripotent state in mouse. In vivo and in vitro counterparts of mouse PSCs and suitable culture conditions are shown. (b) Diagram of the pluripotency state in human development and PSCs with the lineages and signaling pathways to maintain each state described. Blue indicates inhibition. Red indicates activation.

Recently, germ cell competency was analyzed in mouse epiblast. E5.5‒E6.75 mouse epiblast cells could be induced into primordial germ cell (PGC)‐like cells in response to BMP4, whereas those beyond E6.75 could not be induced (Ohinata et al., 2009). The same transient population in vitro, which exists immediately after exiting naïve pluripotency, possesses the capacity to induce PGC‐like cells (Hayashi et al., 2011; Nakamura et al., 2016). This population consists of epiblast‐like cells (EpiLCs), which express *Otx2*, *Oct6*, and *Sox3* and show downregulation of naïve pluripotency‐related genes (*Esrrb*, *Tfcp2l1*, *Klf2*, *Klf4*, and *Rex1*), consistent with the gene expression patterns of E5.5 mouse epiblast (Hayashi et al., 2011, 2012; Ohinata et al., 2009). The pluripotency of EpiLCs was named “formative” pluripotency (Figure 3a) (Kalkan & Smith, 2014; Kinoshita et al., 2018; Smith, 2017). Since primed human PSCs have the ability to differentiate into PGC‐like cells (Irie et al., 2015; Sasaki et al., 2015), the criteria for mouse formative pluripotency cannot be directly applied to that of humans. Nevertheless, formative human PSCs may exist and are considered to represent a period in which the cells gradually acquire the ability to differentiate into somatic and germ cells. After long‐term culturing of naïve PSCs under XAV, FGF2, and Activin A, they transcriptionally resembled primed PSCs and easily differentiated into the three germ layers (Rostovskaya et al., 2019). These capacitated cells may represent human formative PSCs (Rostovskaya et al., 2019), but they could not be maintained as formative stem (FS) cells because of suboptimal culture conditions. In 2020, Kinoshita et al. succeeded to induce and maintain FS cells from mouse and human naïve PSCs and embryos (Figure 3) (Kinoshita et al., 2020). Unlike the induction of capacitated cells, human FS cells require weak activation of TGFβ/Activin signaling and the inhibition of Wnt signaling and retinoic acid receptors (Figure 3b) (Kinoshita et al., 2020). Human FS cells are useful models for analyzing the mechanisms of early embryonic development, especially the acquisition process of the multilineage differentiation potential. They are also useful for optimizing differentiation protocols toward primed pluripotency.

## 5 IN VITRO CULTURES OF MOUSE AND PRIMATE EMBRYOS TOWARD GASTRULATION

Pre‐implantation embryos can be studied relatively easily as far as they can be cultured in vitro. However, the analysis of embryonic development after implantation into the uterus is difficult even in animal models. Therefore, research on in vitro cultures beyond the implantation stage is being conducted. To reproduce implantation in vitro, maternal tissues or alternative materials and culture media for maintaining the embryos beyond implantation are required. Several groups have succeeded in performing 2D embryo cultures until the egg‐cylinder stage in vitro using medium supplemented with serum from calf, rat, or human placental cord blood (Hsu, 1972; Masaki et al., 2015; Morris et al., 2012; Wu et al., 1981). Mouse embryos can grow on a non‐coated plastic plate or on a plate coated with a matrix, such as collagen, fibronectin, or inactivated feeder cells.

In 2014, a new protocol for in vitro embryo culture was developed, which allowed the observation of mouse embryo development from E3.5 to post‐implantation epiblast with an egg‐cylinder‐like structure (Table 3) (Bedzhov et al., 2014). This protocol is based on two media, IVC1 and IVC2, which could be used to culture embryos from humans and cynomolgus monkeys as well as mice (Table 3) (Deglincerti et al., 2016; Niu et al., 2019; Shahbazi et al., 2016; Xiang et al., 2020). The in vitro culture of human embryos could be successfully continued up to 14 days post fertilization, and the cultured embryos demonstrated several post‐implantation events, such as bilaminar disc formation and amniotic cavity formation (Deglincerti et al., 2016). In 2020, further modified culture conditions for human embryos combined with scRNA‐seq revealed the molecular characteristics of early human development (Xiang et al., 2020). Specifically, the study reported the gene expression patterns of the pre‐ and post‐implantation epiblast, primitive endoderm (PrE), amnion, and early primitive streak‐like cells. However, current international guidelines prohibit human embryos from being cultured for more than 14 days or to the time of formation of the primitive streak and gastrula (Hyun et al., 2016, 2020); because no cells from the nervous system appear before this time, embryos have low viability up to this point, and implantation is essential for further development. Instead of human embryos, non‐human primate embryos are applied for embryo cultures of more than 14 days (Ma et al., 2019; Niu et al., 2019). Embryos from cynomolgus monkeys have been cultured up to 20 days from the blastocyst stage to early gastrulation (Table 3) (Ma et al., 2019; Niu et al., 2019). The long‐term embryo culture allowed observation of key developmental events in utero, especially of extraembryonic lineages, such as development of the hypoblast, formation of the amniotic and yolk sac cavities, and development of PGCs. These findings demonstrated that cynomolgus monkey embryos can develop beyond gastrulation in vitro even in the absence of proper maternal tissues. Combining in vitro embryo cultures with endometrial cells may more precisely recapitulate in vivo peri‐implantation development to analyze critical implantation events.

**TABLE 3:** List of methodologies for *in vitro* embryo culture.

Species	Medium	Culture condition	Extracellular matrix	Culture periods	References
Mouse	IVC1, IVC2	Attached	No	d.p.f 5	Bedzhov et al. (2014)
Cynomolgus monkey	IVC1, IVC2 + Y−27632	Attached	No	d.p.f 20	Niu et al. (2019)
	CMRL1066 + FBS, CMRL1066 + KSR, CMRL1066 + Rat serum	Floating	Matrigel	d.p.f 20	Ma et al. (2019)
Human	IVC1, IVC2	Attached	No	d.p.f 12	Deglincerti et al. (2016)
	IVC1, IVC2	Attached	No	d.p.f 13	Shahbazi et al. (2016)
	mIVC1, mIVC2	Floating	Matrigel	d.p.f 14	Xiang et al. (2020)

Note

d.p.f., days post fertilization; mIVC1, IVC1 + sodium lactate and sodium pyruvate; mIVC2, IVC2 + sodium lactate, sodium pyruvate, and Y‐27632.

## 6 RECONSTITUTION OF THE EMBRYO‐LIKE STRUCTURE USING PLURIPOTENT STEM CELLS

Although in vitro embryo cultures provide an advanced method to study peri‐implantation development, they require a large number of human and non‐human primate embryos. Compared to mice, it is difficult to prepare a sufficient number of high‐quality embryos from humans and primates. Moreover, to analyze genetic functions in early embryogenesis, gene knockout is often required. Although genome editing techniques, such as CRISPR, can be applied directly to fertilized primate eggs to induce gene knockouts (Fogarty et al., 2017; Sato et al., 2016), a large number of embryos are required to obtain accurately genome‐edited embryos in addition to the issue of mosaicism. Therefore, unless genome editing improves in terms of accuracy, efficiency, and homogeneity, other methods are needed.

Mouse ESCs have the ability to generate mice composed only of ESC‐derived somatic cells using tetraploid embryos (Nagy et al., 1990; Wang et al., 1997). This ability suggests that it may be possible to form an embryo‐like structure from ESCs in vitro. Such a structure must be capable of differentiating into the three germ layers and mimic the body axis of the natural embryo. Embryoid bodies (EB) can differentiate into the three germ layers and generate embryo‐like structures from floating 3D cultures of PSCs. However, the 3D structures of EBs differ from those of real embryos, suggesting that unidentified factors are needed to regulate the body axis and the proper timing of differentiation.

**FIGURE 4:**
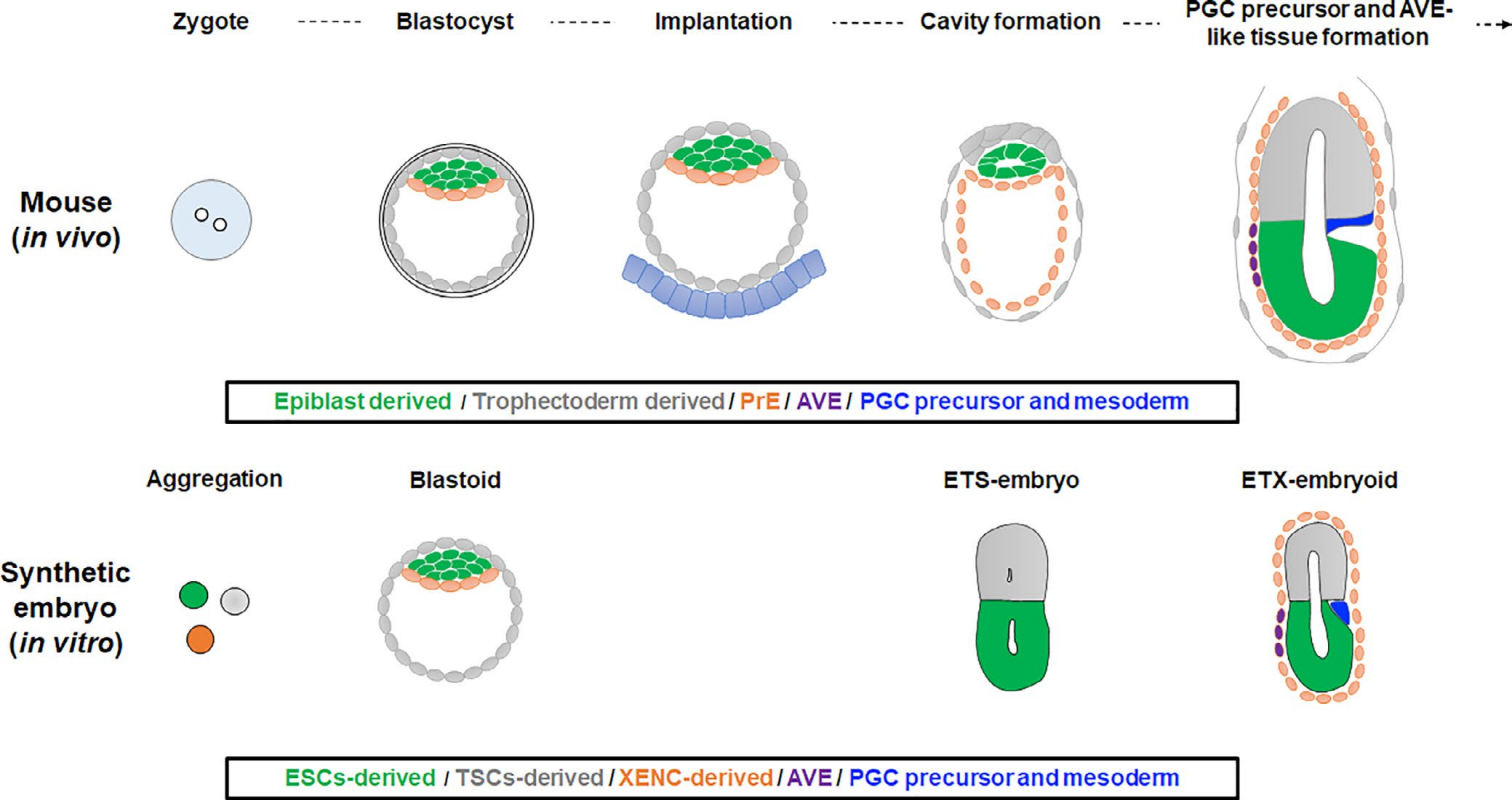
Comparison of natural embryos, blastoids, ETS‐embryos, and ETX‐embryos. Diagram of mouse development from zygote to post‐implantation stages (above) and synthetic embryos corresponding to each of those stages (below).

Harrison et al. developed a co‐culture system that aggregates single ESCs and small clumps of trophectoderm stem cells (TSCs) to form 3D structures called in vitro ESC and TSC stem cell–embryos (ETS‐embryos), which resemble natural embryos (Harrison et al., 2017). ETS‐embryos are highly reproducible and can recapitulate several events of embryo development after implantation, such as cavity formation (Figure 4) (Harrison et al., 2017). On the other hand, ETS‐embryos do not show gastrulation. Normally, a natural embryo in the post‐implantation stage shows PrE, but ETS‐embryos do not have a PrE counterpart. Additionally, ETS‐embryos rely on Matrigel as an extracellular matrix, which is commonly used in organoid cultures, to mimic the PrE counterpart of blastocysts (Harrison et al., 2017). Later, two groups created a culture system composed of ESCs, TSCs, and extraembryonic endoderm stem cells (XENCs) (“ETX‐embryos”), which are derived from the PrE (Sozen et al., 2018; Zhang et al., 2019). The addition of XENCs resulted in a morphology more similar to that of natural mouse embryos and in lumenogenesis, along with the appearance of PGC precursors and formation of anterior visceral endoderm (AVE)‐like tissues, which are not observed in ETS‐embryos (Figure 4) (Sozen et al., 2018; Zhang et al., 2019). These studies revealed that the combination of ESCs and extraembryonic cells can model the properties of pre‐ to post‐implantation embryo, but the initial stages of these embryo‐like structures do not mimic the features of natural embryos, because the ESCs and TSCs are separated.

Another 3D embryo model is the blastocyst‐like structure known as blastoid. A blastoid is formed by aggregating mouse ESCs and TSCs on a microwell plate (Rivron et al., 2018). Unlike ETS‐embryos, blastoids do not require Matrigel. Instead, the microwell induces ESC–TSC aggregation at appropriate cell numbers. The inhibition of Wnt and activation of cAMP signaling pathways further control the organization between the ESCs and TSCs (Rivron et al., 2018). Blastoids consist of ESCs, TSCs, and PDGFRA‐positive PrE cells that are spontaneously differentiated from ESCs. Thus, it is possible that the presence of PrE cells in synthetic embryos does not have a significant effect on the developmental progression up to the blastocyst stage, but it may have an advantage in the proper development and survival of the embryo after implantation. These stem‐cell‐based models are the newest generation for the study of embryogenesis and arguably serve as the best available tools for studying the mechanisms that coordinate embryo morphogenesis and size.

Finally, researchers have generated embryo‐like structures using human PSCs to analyze human peri‐implantation development. Gastruloids are 3D aggregations of primed human PSCs that model post‐gastrulation and are used to observe the elongation of the anteroposterior axis and gene expression profiles during somitogenesis (Moris et al., 2020). However, gastruloids do not model human development at the pre‐implantation stage because primed PSCs are a counterpart of post‐implantation epiblast. Recently, two studies, including one from our group, revealed the differentiation potential of naïve human PSCs into trophectoderm without the forced expression of any transcription factors (Guo et al., 2020; Io et al., 2020). Self‐assembling embryo‐like structures from naïve human PSCs and trophectoderm‐like cells may be able to model human development from the pre‐implantation to the post‐implantation stages.

## 7 CONCLUSIONS

Early developmental studies on humans are limited because of ethical issues. In particular, embryos beyond the implantation stage are not readily accessible. As alternatives, non‐human primate embryos and reconstituted embryos using PSCs are serving as accurate representations of human embryo. In this review, we summarized the current status of mouse and human PSCs, with particular focus on pre‐implantation naïve PSCs to post‐implantation primed PSCs. The formative state is midway between naïve and primed PSCs and bestows the capacity to differentiate into the three germ layers and germ cells. The latest development regarding PSC research for the study of the embryo is the preparation of embryo‐like structures. This approach may enable the best ethically sound study of human post‐implantation embryos. Ultimately, all these efforts are being made to understand the mechanisms of early embryonic development, which defines the growth of the entire body.

## References

[dgd12715-bib-0001] Bedzhov, I. , Leung, C. Y. , Bialecka, M. , & Zernicka‐Goetz, M. (2014, Dec). In vitro culture of mouse blastocysts beyond the implantation stages. Nat Protoc, 9(12), 2732–2739. 10.1038/nprot.2014.186 25356584

[dgd12715-bib-0002] Blakeley, P. , Fogarty, N. M. , del Valle, I. , Wamaitha, S. E. , Hu, T. X. , Elder, K. , Snell, P. , Christie, L. , Robson, P. , & Niakan, K. K. (2015, Sep 15). Defining the three cell lineages of the human blastocyst by single‐cell RNA‐seq. Development, 142(18), 3151–3165. 10.1242/dev.123547 26293300 PMC4582176

[dgd12715-bib-0003] Boroviak, T. , & Nichols, J. (2014, Dec 5). The birth of embryonic pluripotency. Philos Trans RSocof Lond B Biol Sci, 369(1657), 10.1098/rstb.2013.0541 PMC4216464 25349450

[dgd12715-bib-0004] Boroviak, T. , & Nichols, J. (2017, Jan 15). Primate embryogenesis predicts the hallmarks of human naive pluripotency. Development, 144(2), 175–186. 10.1242/dev.145177 28096211 PMC5430762

[dgd12715-bib-0005] Boroviak, T. , Stirparo, G. G. , Dietmann, S. , Hernando‐Herraez, I. , Mohammed, H. , Reik, W. , Smith, A. , Sasaki, E. , Nichols, J. , & Bertone, P. (2018, Nov 9). Single cell transcriptome analysis of human, marmoset and mouse embryos reveals common and divergent features of preimplantation development. Development, 145(21), 10.1242/dev.167833 PMC6240320 30413530

[dgd12715-bib-0006] Bredenkamp, N. , Stirparo, G. G. , Nichols, J. , Smith, A. , & Guo, G. (2019, Jun 11). The cell‐surface marker sushi containing domain 2 facilitates establishment of human naive pluripotent stem cells. Stem Cell Rep, 12(6), 1212–1222. 10.1016/j.stemcr.2019.03.014 PMC6565611 31031191

[dgd12715-bib-0007] Brons, I. G. , Smithers, L. E. , Trotter, M. W. , Rugg‐Gunn, P. , Sun, B. , de Sousa, C. , Lopes, S. M. , Howlett, S. K. , Clarkson, A. , Ahrlund‐Richter, L. , Pedersen, R. A. , & Vallier, L. (2007, Jul 12). Derivation of pluripotent epiblast stem cells from mammalian embryos. Nature, 448(7150), 191–195. 10.1038/nature05950 17597762

[dgd12715-bib-0008] Buehr, M. , Meek, S. , Blair, K. , Yang, J. , Ure, J. , Silva, J. , McLay, R. , Hall, J. , Ying, Q. L. , & Smith, A. (2008, Dec 26). Capture of authentic embryonic stem cells from rat blastocysts. Cell, 135(7), 1287–1298. 10.1016/j.cell.2008.12.007 19109897

[dgd12715-bib-0009] Chan, Y. S. , Goke, J. , Ng, J. H. , Lu, X. , Gonzales, K. A. , Tan, C. P. , Tng, W. Q. , Hong, Z. Z. , Lim, Y. S. , & Ng, H. H. (2013, Dec 5). Induction of a human pluripotent state with distinct regulatory circuitry that resembles preimplantation epiblast. Cell Stem Cell, 13(6), 663–675. 10.1016/j.stem.2013.11.015 24315441

[dgd12715-bib-0010] Chen, D. , Liu, W. , Zimmerman, J. , Pastor, W. A. , Kim, R. , Hosohama, L. , Ho, J. , Aslanyan, M. , Gell, J. J. , Jacobsen, S. E. , & Clark, A. T. (2018, Dec 26). The TFAP2C‐regulated OCT4 naive enhancer is involved in human germline formation. Cell Rep, 25(13), 3591–3602 e3595. 10.1016/j.celrep.2018.12.011 30590035 PMC6342560

[dgd12715-bib-0011] Chen, H. , Aksoy, I. , Gonnot, F. , Osteil, P. , Aubry, M. , Hamela, C. , Rognard, C. , Hochard, A. , Voisin, S. , Fontaine, E. , Mure, M. , Afanassieff, M. , Cleroux, E. , Guibert, S. , Chen, J. , Vallot, C. , Acloque, H. , Genthon, C. , & Donnadieu, C. , … Savatier, P. (2015, May 13). Reinforcement of STAT3 activity reprogrammes human embryonic stem cells to naive‐like pluripotency. Nat Commun, 6(1), 7095. 10.1038/ncomms8095 25968054 PMC4479042

[dgd12715-bib-0012] Collier, A. J. , Panula, S. P. , Schell, J. P. , Chovanec, P. , Plaza Reyes, A. , Petropoulos, S. , Corcoran, A. E. , Walker, R. , Douagi, I. , Lanner, F. , & Rugg‐Gunn, P. J. (2017, Jun 1). Comprehensive cell surface protein profiling identifies specific markers of human naive and primed pluripotent states. Cell Stem Cell, 20(6), 874–890 e877. 10.1016/j.stem.2017.02.014 28343983 PMC5459756

[dgd12715-bib-0013] Deglincerti, A. , Croft, G. F. , Pietila, L. N. , Zernicka‐Goetz, M. , Siggia, E. D. , & Brivanlou, A. H. (2016, May 12). Self‐organization of the in vitro attached human embryo. Nature, 533(7602), 251–254. 10.1038/nature17948 27144363

[dgd12715-bib-0014] Di Stefano, B. , Ueda, M. , Sabri, S. , Brumbaugh, J. , Huebner, A. J. , Sahakyan, A. , Clement, K. , Clowers, K. J. , Erickson, A. R. , Shioda, K. , Gygi, S. P. , Gu, H. , Shioda, T. , Meissner, A. , Takashima, Y. , Plath, K. , & Hochedlinger, K. (2018, Aug 20). Reduced MEK inhibition preserves genomic stability in naive human embryonic stem cells. Nat Methods, 15(9), 732–740. 10.1038/s41592-018-0104-1 30127506 PMC6127858

[dgd12715-bib-0015] Evans, M. J. , & Kaufman, M. H. (1981, Jul 9). Establishment in culture of pluripotential cells from mouse embryos. Nature, 292(5819), 154–156. 7242681 10.1038/292154a0

[dgd12715-bib-0016] Festuccia, N. , Osorno, R. , Halbritter, F. , Karwacki‐Neisius, V. , Navarro, P. , Colby, D. , Wong, F. , Yates, A. , Tomlinson, S. R. , & Chambers, I. (2012, Oct 5). Esrrb is a direct Nanog target gene that can substitute for Nanog function in pluripotent cells. Cell Stem Cell, 11(4), 477–490. 10.1016/j.stem.2012.08.002 23040477 PMC3473361

[dgd12715-bib-0017] Fogarty, N. M. E. , McCarthy, A. , Snijders, K. E. , Powell, B. E. , Kubikova, N. , Blakeley, P. , Lea, R. , Elder, K. , Wamaitha, S. E. , Kim, D. , Maciulyte, V. , Kleinjung, J. , Kim, J. S. , Wells, D. , Vallier, L. , Bertero, A. , Turner, J. M. A. , & Niakan, K. K. (2017, Oct 5). Genome editing reveals a role for OCT4 in human embryogenesis. Nature, 550(7674), 67–73. 10.1038/nature24033 28953884 PMC5815497

[dgd12715-bib-0018] Fort, A. , Hashimoto, K. , Yamada, D. , Salimullah, M. D. , Keya, C. A. , Saxena, A. , Bonetti, A. , Voineagu, I. , Bertin, N. , Kratz, A. , Noro, Y. , Wong, C.‐H. , de Hoon, M. , Andersson, R. , Sandelin, A. , Suzuki, H. , Wei, C.‐L. , Koseki, H. , Hasegawa, Y. , … Carninci, P. (2014). Jun). Deep transcriptome profiling of mammalian stem cells supports a regulatory role for retrotransposons in pluripotency maintenance. Nat Genet, 46(6), 558–566. 10.1038/ng.2965 24777452

[dgd12715-bib-0019] Gafni, O. , Weinberger, L. , Mansour, A. A. , Manor, Y. S. , Chomsky, E. , Ben‐Yosef, D. , Kalma, Y. , Viukov, S. , Maza, I. , Zviran, A. , Rais, Y. , Shipony, Z. , Mukamel, Z. , Krupalnik, V. , Zerbib, M. , Geula, S. , Caspi, I. , Schneir, D. , Shwartz, T. , … Hanna, J. H. (2013, Dec 12). Derivation of novel human ground state naive pluripotent stem cells. Nature, 504(7479), 282–286. 10.1038/nature12745 24172903

[dgd12715-bib-0020] Goke, J. , Lu, X. , Chan, Y. S. , Ng, H. H. , Ly, L. H. , Sachs, F. , & Szczerbinska, I. (2015, Feb 5). Dynamic transcription of distinct classes of endogenous retroviral elements marks specific populations of early human embryonic cells. Cell Stem Cell, 16(2), 135–141. 10.1016/j.stem.2015.01.005 25658370

[dgd12715-bib-0021] Guo, G. , & Smith, A. (2010, Oct). A genome‐wide screen in EpiSCs identifies Nr5a nuclear receptors as potent inducers of ground state pluripotency. Development, 137(19), 3185–3192. 10.1242/dev.052753 20823062 PMC2934732

[dgd12715-bib-0022] Guo, G. , Stirparo, G. G. , Strawbridge, S. , Spindlow, D. , Yang, J. , Clarke, J. , Dattani, A. , Yanagida, A. , Li, M. A. , Myers, S. , Özel, B. N. , Nichols, J. , & Smith, A. (2020). Human naïve epiblast cells possess unrestricted lineage potential. BioRxiv, 2020.2002.2004.933812. 10.1101/2020.02.04.933812 PMC8189439 33831366

[dgd12715-bib-0023] Guo, G. , von Meyenn, F. , Rostovskaya, M. , Clarke, J. , Dietmann, S. , Baker, D. , Sahakyan, A. , Myers, S. , Bertone, P. , Reik, W. , Plath, K. , & Smith, A. (2017, Aug 1). Epigenetic resetting of human pluripotency. Development, 144(15), 2748–2763. 10.1242/dev.146811 28765214 PMC5560041

[dgd12715-bib-0024] Guo, G. , von Meyenn, F. , Santos, F. , Chen, Y. , Reik, W. , Bertone, P. , Smith, A. , & Nichols, J. (2016, Apr 12). Naive pluripotent stem cells derived directly from isolated cells of the human inner cell mass. Stem Cell Rep, 6(4), 437–446. 10.1016/j.stemcr.2016.02.005 PMC4834040 26947977

[dgd12715-bib-0025] Guo, G. , Yang, J. , Nichols, J. , Hall, J. S. , Eyres, I. , Mansfield, W. , & Smith, A. (2009, Apr). Klf4 reverts developmentally programmed restriction of ground state pluripotency. Development, 136(7), 1063–1069. 10.1242/dev.030957 19224983 PMC2685927

[dgd12715-bib-0026] Hall, J. , Guo, G. , Wray, J. , Eyres, I. , Nichols, J. , Grotewold, L. , Morfopoulou, S. , Humphreys, P. , Mansfield, W. , Walker, R. , Tomlinson, S. , & Smith, A. (2009, Dec 4). Oct4 and LIF/Stat3 additively induce Kruppel factors to sustain embryonic stem cell self‐renewal. Cell Stem Cell, 5(6), 597–609. 10.1016/j.stem.2009.11.003 19951688

[dgd12715-bib-0027] Han, D. W. , Tapia, N. , Joo, J. Y. , Greber, B. , Arauzo‐Bravo, M. J. , Bernemann, C. , Ko, K. , Wu, G. , Stehling, M. , Do, J. T. , & Scholer, H. R. (2010, Nov 12). Epiblast stem cell subpopulations represent mouse embryos of distinct pregastrulation stages. Cell, 143(4), 617–627. 10.1016/j.cell.2010.10.015 21056461

[dgd12715-bib-0028] Hanna, J. , Cheng, A. W. , Saha, K. , Kim, J. , Lengner, C. J. , Soldner, F. , Cassady, J. P. , Muffat, J. , Carey, B. W. , & Jaenisch, R. (2010, May 18). Human embryonic stem cells with biological and epigenetic characteristics similar to those of mouse ESCs. Proc Natl Acad Sci U S A, 107(20), 9222–9227. 10.1073/pnas.1004584107 20442331 PMC2889088

[dgd12715-bib-0029] Harrison, S. E. , Sozen, B. , Christodoulou, N. , Kyprianou, C. , & Zernicka‐Goetz, M. (2017, Apr 14). Assembly of embryonic and extraembryonic stem cells to mimic embryogenesis in vitro. Science, 356, 6334. 10.1126/science.aal1810 28254784

[dgd12715-bib-0030] Hayashi, K. , Ogushi, S. , Kurimoto, K. , Shimamoto, S. , Ohta, H. , & Saitou, M. (2012, Nov 16). Offspring from oocytes derived from in vitro primordial germ cell‐like cells in mice. Science, 338(6109), 971–975. 10.1126/science.1226889 23042295

[dgd12715-bib-0031] Hayashi, K. , Ohta, H. , Kurimoto, K. , Aramaki, S. , & Saitou, M. (2011, Aug 19). Reconstitution of the mouse germ cell specification pathway in culture by pluripotent stem cells. Cell, 146(4), 519–532. 10.1016/j.cell.2011.06.052 21820164

[dgd12715-bib-0032] Hertig, A. T. , & Rock, J. (1949, Feb). Two human ova of the pre‐villous stage, having a developmental age of about 8 and 9 days respectively. Contrib Embryol, 33(213–221), 169–186. 18130387

[dgd12715-bib-0033] Hotta, A. , Cheung, A. Y. , Farra, N. , Vijayaragavan, K. , Seguin, C. A. , Draper, J. S. , Pasceri, P. , Maksakova, I. A. , Mager, D. L. , Rossant, J. , Bhatia, M. , & Ellis, J. (2009, May). Isolation of human iPS cells using EOS lentiviral vectors to select for pluripotency. Nat Methods, 6(5), 370–376. 10.1038/nmeth.1325 19404254

[dgd12715-bib-0034] Hsu, Y. C. (1972, Sep 22). Differentiation in vitro of mouse embryos beyond the implantation stage. Nature, 239(5369), 200–202. 10.1038/239200a0 4562729

[dgd12715-bib-0035] Huang, K. , Maruyama, T. , & Fan, G. (2014, Oct 2). The naive state of human pluripotent stem cells: a synthesis of stem cell and preimplantation embryo transcriptome analyses. Cell Stem Cell, 15(4), 410–415. 10.1016/j.stem.2014.09.014 25280217 PMC5507179

[dgd12715-bib-0036] Hyun, I. , Munsie, M. , Pera, M. F. , Rivron, N. C. , & Rossant, J. (2020, Feb 11). Toward guidelines for research on human embryo models formed from stem cells. Stem Cell Rep, 14(2), 169–174. 10.1016/j.stemcr.2019.12.008 PMC7015820 31951813

[dgd12715-bib-0037] Hyun, I. , Wilkerson, A. , & Johnston, J. (2016, May 12). Embryology policy: revisit the 14‐day rule. Nature, 533(7602), 169–171. 10.1038/533169a 27172031

[dgd12715-bib-0038] Io, S. , Kabata, M. , Iemura, Y. , Semi, K. , Morone, N. , Okamoto, I. , Nakamura, T. , Kojima, Y. , Iwatani, C. , Tsuchiya, H. , Kaswandy, B. , Kondoh, E. , Saitou, M. , Yamamoto, T. , Mandai, M. , & Takashima, Y. (2020). Capturing human trophoblast development with naïve pluripotent stem cells in vitro. BioRxiv, 2020.2012.2017.416800. 10.1101/2020.12.17.416800 33831365

[dgd12715-bib-0039] Irie, N. , Weinberger, L. , Tang, W. W. , Kobayashi, T. , Viukov, S. , Manor, Y. S. , Dietmann, S. , Hanna, J. H. , & Surani, M. A. (2015, Jan 15). SOX17 is a critical specifier of human primordial germ cell fate. Cell, 160(1–2), 253–268. 10.1016/j.cell.2014.12.013 25543152 PMC4310934

[dgd12715-bib-0040] Kalkan, T. , & Smith, A. (2014, Dec 5). Mapping the route from naive pluripotency to lineage specification. Philos Trans R Soc Lond B Biol Sci, 369(1657), 10.1098/rstb.2013.0540 PMC4216463 25349449

[dgd12715-bib-0041] Kinoshita, M. , Barber, M. , Mansfield, W. , Cui, Y. , Spindlow, D. , Stirparo, G. G. , Dietmann, S. , Nichols, J. , & Smith, A. (2020, Nov 24). Capture of mouse and human stem cells with features of formative pluripotency. Cell Stem Cell, 10.1016/j.stem.2020.11.005 PMC8657791 34861148

[dgd12715-bib-0042] Kinoshita, M. , & Smith, A. (2018, Jan). Pluripotency deconstructed. Dev Growth Differ, 60(1), 44–52. 10.1111/dgd.12419 29359419

[dgd12715-bib-0043] Li, P. , Tong, C. , Mehrian‐Shai, R. , Jia, L. , Wu, N. , Yan, Y. , Maxson, R. E. , Schulze, E. N. , Song, H. , Hsieh, C. L. , Pera, M. F. , & Ying, Q. L. (2008, Dec 26). Germline competent embryonic stem cells derived from rat blastocysts. Cell, 135(7), 1299–1310. 10.1016/j.cell.2008.12.006 19109898 PMC2735113

[dgd12715-bib-0044] Liu, X. , Nefzger, C. M. , Rossello, F. J. , Chen, J. , Knaupp, A. S. , Firas, J. , Ford, E. , Pflueger, J. , Paynter, J. M. , Chy, H. S. , O'Brien, C. M. , Huang, C. , Mishra, K. , Hodgson‐Garms, M. , Jansz, N. , Williams, S. M. , Blewitt, M. E. , Nilsson, S. K. , Schittenhelm, R. B. , … Polo, J. M. (2017, Nov). Comprehensive characterization of distinct states of human naive pluripotency generated by reprogramming. Nat Methods, 14(11), 1055–1062. 10.1038/nmeth.4436 28945704

[dgd12715-bib-0045] Lu, X. , Sachs, F. , Ramsay, L. , Jacques, P. E. , Goke, J. , Bourque, G. , & Ng, H. H. (2014, Apr). The retrovirus HERVH is a long noncoding RNA required for human embryonic stem cell identity. Nat Struct Mol Biol, 21(4), 423–425. 10.1038/nsmb.2799 24681886

[dgd12715-bib-0046] Ludwig, T. E. , Levenstein, M. E. , Jones, J. M. , Berggren, W. T. , Mitchen, E. R. , Frane, J. L. , Crandall, L. J. , Daigh, C. A. , Conard, K. R. , Piekarczyk, M. S. , Llanas, R. A. , & Thomson, J. A. (2006, Feb). Derivation of human embryonic stem cells in defined conditions. Nat Biotechnol, 24(2), 185–187. 10.1038/nbt1177 16388305

[dgd12715-bib-0047] Ma, H. , Zhai, J. , Wan, H. , Jiang, X. , Wang, X. , Wang, L. , Xiang, Y. , He, X. , Zhao, Z. A. , Zhao, B. , Zheng, P. , Li, L. , & Wang, H. (2019, Nov 15). In vitro culture of cynomolgus monkey embryos beyond early gastrulation. Science, 366(6467), 10.1126/science.aax7890 31672918

[dgd12715-bib-0048] Maksakova, I. A. , & Mager, D. L. (2005, Nov). Transcriptional regulation of early transposon elements, an active family of mouse long terminal repeat retrotransposons. J Virol, 79(22), 13865–13874. 10.1128/JVI.79.22.13865-13874.2005 16254322 PMC1280189

[dgd12715-bib-0049] Martin, G. R. (1981, Dec). Isolation of a pluripotent cell line from early mouse embryos cultured in medium conditioned by teratocarcinoma stem cells. Proc Natl Acad Sci U S A, 78(12), 7634–7638. 10.1073/pnas.78.12.7634 6950406 PMC349323

[dgd12715-bib-0050] Masaki, H. , Kato‐Itoh, M. , Umino, A. , Sato, H. , Hamanaka, S. , Kobayashi, T. , Yamaguchi, T. , Nishimura, K. , Ohtaka, M. , Nakanishi, M. , & Nakauchi, H. (2015, Sep 15). Interspecific in vitro assay for the chimera‐forming ability of human pluripotent stem cells. Development, 142(18), 3222–3230. 10.1242/dev.124016 26023098

[dgd12715-bib-0051] Moris, N. , Anlas, K. , van den Brink, S. C. , Alemany, A. , Schroder, J. , Ghimire, S. , Balayo, T. , van Oudenaarden, A. , & Martinez Arias, A. (2020, Jun). An in vitro model of early anteroposterior organization during human development. Nature, 582(7812), 410–415. 10.1038/s41586-020-2383-9 32528178

[dgd12715-bib-0052] Morris, S. A. , Grewal, S. , Barrios, F. , Patankar, S. N. , Strauss, B. , Buttery, L. , Alexander, M. , Shakesheff, K. M. , & Zernicka‐Goetz, M. (2012, Feb 14). Dynamics of anterior‐posterior axis formation in the developing mouse embryo. Nat Commun, 3, 673. 10.1038/ncomms1671 22334076 PMC3293425

[dgd12715-bib-0053] Nagy, A. , Gocza, E. , Diaz, E. M. , Prideaux, V. R. , Ivanyi, E. , Markkula, M. , & Rossant, J. (1990, Nov). Embryonic stem cells alone are able to support fetal development in the mouse. Development, 110(3), 815–821. 2088722 10.1242/dev.110.3.815

[dgd12715-bib-0054] Nakamura, T. , Okamoto, I. , Sasaki, K. , Yabuta, Y. , Iwatani, C. , Tsuchiya, H. , Seita, Y. , Nakamura, S. , Yamamoto, T. , & Saitou, M. (2016, Sep 1). A developmental coordinate of pluripotency among mice, monkeys and humans. Nature, 537(7618), 57–62. 10.1038/nature19096 27556940

[dgd12715-bib-0055] Nichols, J. , Jones, K. , Phillips, J. M. , Newland, S. A. , Roode, M. , Mansfield, W. , Smith, A. , & Cooke, A. (2009, Jul). Validated germline‐competent embryonic stem cell lines from nonobese diabetic mice. Nat Med, 15(7), 814–818. 10.1038/nm.1996 19491843

[dgd12715-bib-0056] Nichols, J. , & Smith, A. (2009, Jun 5). Naive and primed pluripotent states. Cell Stem Cell, 4(6), 487–492. 10.1016/j.stem.2009.05.015 19497275

[dgd12715-bib-0057] Niu, Y. , Sun, N. , Li, C. , Lei, Y. , Huang, Z. , Wu, J. , Si, C. , Dai, X. , Liu, C. , Wei, J. , Liu, L. , Feng, S. , Kang, Y. , Si, W. , Wang, H. , Zhang, E. , Zhao, L. , Li, Z. , Luo, X. , … Tan, T. (2019, Nov 15). Dissecting primate early post‐implantation development using long‐term in vitro embryo culture. Science, 366(6467), 10.1126/science.aaw5754 31672917

[dgd12715-bib-0058] O’Rahilly, R. , & Muller, F. (1987). Development Stages in Human Embryos: Including a Revision of Streeter’s “Horizons” and a Survey of the Carnegie Collection. Carnegie Institution of Washington. https://www.ehd.org/developmental‐stages/stage0.php

[dgd12715-bib-0059] Ohinata, Y. , Ohta, H. , Shigeta, M. , Yamanaka, K. , Wakayama, T. , & Saitou, M. (2009, May 1). A signaling principle for the specification of the germ cell lineage in mice. Cell, 137(3), 571–584. 10.1016/j.cell.2009.03.014 19410550

[dgd12715-bib-0060] Ohnuki, M. , Tanabe, K. , Sutou, K. , Teramoto, I. , Sawamura, Y. , Narita, M. , Nakamura, M. , Tokunaga, Y. , Nakamura, M. , Watanabe, A. , Yamanaka, S. , & Takahashi, K. (2014, Aug 26). Dynamic regulation of human endogenous retroviruses mediates factor‐induced reprogramming and differentiation potential. Proc Natl Acad Sci U S A, 111(34), 12426–12431. 10.1073/pnas.1413299111 25097266 PMC4151758

[dgd12715-bib-0061] Pastor, W. A. , Liu, W. , Chen, D. , Ho, J. , Kim, R. , Hunt, T. J. , Lukianchikov, A. , Liu, X. , Polo, J. M. , Jacobsen, S. E. , & Clark, A. T. (2018, May). TFAP2C regulates transcription in human naive pluripotency by opening enhancers. Nat Cell Biol, 20(5), 553–564. 10.1038/s41556-018-0089-0 29695788 PMC5926822

[dgd12715-bib-0062] Qin, H. , Hejna, M. , Liu, Y. , Percharde, M. , Wossidlo, M. , Blouin, L. , Durruthy‐Durruthy, J. , Wong, P. , Qi, Z. , Yu, J. , Qi, L. S. , Sebastiano, V. , Song, J. S. , & Ramalho‐Santos, M. (2016, Mar 15). YAP induces human naive pluripotency. Cell Rep, 14(10), 2301–2312. 10.1016/j.celrep.2016.02.036 26947063 PMC4807727

[dgd12715-bib-0063] Rivron, N. C. , Frias‐Aldeguer, J. , Vrij, E. J. , Boisset, J. C. , Korving, J. , Vivie, J. , Truckenmuller, R. K. , van Oudenaarden, A. , van Blitterswijk, C. A. , & Geijsen, N. (2018, May). Blastocyst‐like structures generated solely from stem cells. Nature, 557(7703), 106–111. 10.1038/s41586-018-0051-0 29720634

[dgd12715-bib-0064] Rostovskaya, M. , Stirparo, G. G. , & Smith, A. (2019, Apr 3). Capacitation of human naive pluripotent stem cells for multi‐lineage differentiation. Development, 146(7), 10.1242/dev.172916 PMC6467473 30944104

[dgd12715-bib-0065] Sasaki, K. , Yokobayashi, S. , Nakamura, T. , Okamoto, I. , Yabuta, Y. , Kurimoto, K. , Ohta, H. , Moritoki, Y. , Iwatani, C. , Tsuchiya, H. , Nakamura, S. , Sekiguchi, K. , Sakuma, T. , Yamamoto, T. , Mori, T. , Woltjen, K. , Nakagawa, M. , Yamamoto, T. , Takahashi, K. , … Saitou, M. (2015, Aug 6). Robust in vitro induction of human germ cell fate from pluripotent stem cells. Cell Stem Cell, 17(2), 178–194. 10.1016/j.stem.2015.06.014 26189426

[dgd12715-bib-0066] Sato, K. , Oiwa, R. , Kumita, W. , Henry, R. , Sakuma, T. , Ito, R. , Nozu, R. , Inoue, T. , Katano, I. , Sato, K. , Okahara, N. , Okahara, J. , Shimizu, Y. , Yamamoto, M. , Hanazawa, K. , Kawakami, T. , Kametani, Y. , Suzuki, R. , Takahashi, T. , … Sasaki, E. (2016, Jul 7). Generation of a nonhuman primate model of severe combined immunodeficiency using highly efficient genome editing. Cell Stem Cell, 19(1), 127–138. 10.1016/j.stem.2016.06.003 27374787

[dgd12715-bib-0067] Shahbazi, M. N. , Jedrusik, A. , Vuoristo, S. , Recher, G. , Hupalowska, A. , Bolton, V. , Fogarty, N. N. M. , Campbell, A. , Devito, L. , Ilic, D. , Khalaf, Y. , Niakan, K. K. , Fishel, S. , & Zernicka‐Goetz, M. (2016. Jun). Self‐organization of the human embryo in the absence of maternal tissues. Nat Cell Biol, 18(6), 700–708. 10.1038/ncb3347 27144686 PMC5049689

[dgd12715-bib-0068] Silva, J. , Nichols, J. , Theunissen, T. W. , Guo, G. , van Oosten, A. L. , Barrandon, O. , Wray, J. , Yamanaka, S. , Chambers, I. , & Smith, A. (2009, Aug 21). Nanog is the gateway to the pluripotent ground state. Cell, 138(4), 722–737. 10.1016/j.cell.2009.07.039 19703398 PMC3437554

[dgd12715-bib-0069] Smith, A. (2017, Feb 1). Formative pluripotency: the executive phase in a developmental continuum. Development, 144(3), 365–373. 10.1242/dev.142679 28143843 PMC5430734

[dgd12715-bib-0070] Smith, A. G. , Heath, J. K. , Donaldson, D. D. , Wong, G. G. , Moreau, J. , Stahl, M. , & Rogers, D. (1988, Dec 15). Inhibition of pluripotential embryonic stem cell differentiation by purified polypeptides. Nature, 336(6200), 688–690. 10.1038/336688a0 3143917

[dgd12715-bib-0071] Sozen, B. , Amadei, G. , Cox, A. , Wang, R. , Na, E. , Czukiewska, S. , Chappell, L. , Voet, T. , Michel, G. , Jing, N. , Glover, D. M. , & Zernicka‐Goetz, M. (2018, Aug). Self‐assembly of embryonic and two extra‐embryonic stem cell types into gastrulating embryo‐like structures. Nat Cell Biol, 20(8), 979–989. 10.1038/s41556-018-0147-7 30038254

[dgd12715-bib-0072] Stirparo, G. G. , Boroviak, T. , Guo, G. , Nichols, J. , Smith, A. , & Bertone, P. (2018, Feb 7). Integrated analysis of single‐cell embryo data yields a unified transcriptome signature for the human pre‐implantation epiblast. Development, 145(3), 10.1242/dev.158501 PMC5818005 29361568

[dgd12715-bib-0073] Szczerbinska, I. , Gonzales, K. A. U. , Cukuroglu, E. , Ramli, M. N. B. , Lee, B. P. G. , Tan, C. P. , Wong, C. K. , Rancati, G. I. , Liang, H. , Goke, J. , Ng, H. H. , & Chan, Y. S. (2019, Oct 8). A chemically defined feeder‐free system for the establishment and maintenance of the human naive pluripotent state. Stem Cell Rep, 13(4), 612–626. 10.1016/j.stemcr.2019.08.005 PMC6829768 31522974

[dgd12715-bib-0074] Takahashi, K. , & Yamanaka, S. (2006, Aug 25). Induction of pluripotent stem cells from mouse embryonic and adult fibroblast cultures by defined factors. Cell, 126(4), 663–676. 10.1016/j.cell.2006.07.024 16904174

[dgd12715-bib-0075] Takashima, Y. , Guo, G. , Loos, R. , Nichols, J. , Ficz, G. , Krueger, F. , Oxley, D. , Santos, F. , Clarke, J. , Mansfield, W. , Reik, W. , Bertone, P. , & Smith, A. (2014, Sep 11). Resetting transcription factor control circuitry toward ground‐state pluripotency in human. Cell, 158(6), 1254–1269. 10.1016/j.cell.2014.08.029 25215486 PMC4162745

[dgd12715-bib-0076] Tesar, P. J. , Chenoweth, J. G. , Brook, F. A. , Davies, T. J. , Evans, E. P. , Mack, D. L. , Gardner, R. L. , & McKay, R. D. (2007, Jul 12). New cell lines from mouse epiblast share defining features with human embryonic stem cells. Nature, 448(7150), 196–199. 10.1038/nature05972 17597760

[dgd12715-bib-0077] Theunissen, T. W. , Friedli, M. , He, Y. , Planet, E. , O'Neil, R. C. , Markoulaki, S. , Pontis, J. , Wang, H. , Iouranova, A. , Imbeault, M. , Duc, J. , Cohen, M. A. , Wert, K. J. , Castanon, R. , Zhang, Z. , Huang, Y. , Nery, J. R. , Drotar, J. , Lungjangwa, T. , … Jaenisch, R. (2016, Oct 6). Molecular criteria for defining the naive human pluripotent state. Cell Stem Cell, 19(4), 502–515. 10.1016/j.stem.2016.06.011 27424783 PMC5065525

[dgd12715-bib-0078] Theunissen, T. W. , Powell, B. E. , Wang, H. , Mitalipova, M. , Faddah, D. A. , Reddy, J. , Fan, Z. P. , Maetzel, D. , Ganz, K. , Shi, L. , Lungjangwa, T. , Imsoonthornruksa, S. , Stelzer, Y. , Rangarajan, S. , D'Alessio, A. , Zhang, J. , Gao, Q. , Dawlaty, M. M. , Young, R. A. , … Jaenisch, R. (2014, Oct 2). Systematic identification of culture conditions for induction and maintenance of naive human pluripotency. Cell Stem Cell, 15(4), 471–487. 10.1016/j.stem.2014.07.002 25090446 PMC4184977

[dgd12715-bib-0079] Thomson, J. A. , Itskovitz‐Eldor, J. , Shapiro, S. S. , Waknitz, M. A. , Swiergiel, J. J. , Marshall, V. S. , & Jones, J. M. (1998, Nov 6). Embryonic stem cell lines derived from human blastocysts. Science, 282(5391), 1145–1147. 9804556 10.1126/science.282.5391.1145

[dgd12715-bib-0080] Vallier, L. , Alexander, M. , & Pedersen, R. A. (2005, Oct 1). Activin/Nodal and FGF pathways cooperate to maintain pluripotency of human embryonic stem cells. J Cell Sci, 118(Pt 19), 4495–4509. 10.1242/jcs.02553 16179608

[dgd12715-bib-0081] Wang, J. , Xie, G. , Singh, M. , Ghanbarian, A. T. , Rasko, T. , Szvetnik, A. , Cai, H. , Besser, D. , Prigione, A. , Fuchs, N. V. , Schumann, G. G. , Chen, W. , Lorincz, M. C. , Ivics, Z. , Hurst, L. D. , & Izsvak, Z. (2014, Dec 18). Primate‐specific endogenous retrovirus‐driven transcription defines naive‐like stem cells. Nature, 516(7531), 405–409. 10.1038/nature13804 25317556

[dgd12715-bib-0082] Wang, W. , Yang, J. , Liu, H. , Lu, D. , Chen, X. , Zenonos, Z. , Campos, L. S. , Rad, R. , Guo, G. , Zhang, S. , Bradley, A. , & Liu, P. (2011, Nov 8). Rapid and efficient reprogramming of somatic cells to induced pluripotent stem cells by retinoic acid receptor gamma and liver receptor homolog 1. Proc Natl Acad Sci U S A, 108(45), 18283–18288. 10.1073/pnas.1100893108 21990348 PMC3215025

[dgd12715-bib-0083] Wang, Z. Q. , Kiefer, F. , Urbanek, P. , & Wagner, E. F. (1997, Mar). Generation of completely embryonic stem cell‐derived mutant mice using tetraploid blastocyst injection. Mech Dev, 62(2), 137–145. 10.1016/s0925-4773(97)00655-2 9152006

[dgd12715-bib-0084] Ware, C. B. , Nelson, A. M. , Mecham, B. , Hesson, J. , Zhou, W. , Jonlin, E. C. , Jimenez‐Caliani, A. J. , Deng, X. , Cavanaugh, C. , Cook, S. , Tesar, P. J. , Okada, J. , Margaretha, L. , Sperber, H. , Choi, M. , Blau, C. A. , Treuting, P. M. , Hawkins, R. D. , Cirulli, V. , & Ruohola‐Baker, H. (2014, Mar 25). Derivation of naive human embryonic stem cells. Proc Natl Acad Sci U S A, 111(12), 4484–4489. 10.1073/pnas.1319738111 24623855 PMC3970494

[dgd12715-bib-0085] Williams, R. L. , Hilton, D. J. , Pease, S. , Willson, T. A. , Stewart, C. L. , Gearing, D. P. , Wagner, E. F. , Metcalf, D. , Nicola, N. A. , & Gough, N. M. (1988, Dec 15). Myeloid leukaemia inhibitory factor maintains the developmental potential of embryonic stem cells. Nature, 336(6200), 684–687. 10.1038/336684a0 3143916

[dgd12715-bib-0086] Wu, T. C. , Wan, Y. J. , & Damjanov, I. (1981, Oct). Positioning of inner cell mass determines the development of mouse blastocysts in vitro. J Embryol Exp Morphol, 65, 105–117. 7334294

[dgd12715-bib-0087] Xiang, L. , Yin, Y. , Zheng, Y. , Ma, Y. , Li, Y. , Zhao, Z. , Guo, J. , Ai, Z. , Niu, Y. , Duan, K. , He, J. , Ren, S. , Wu, D. , Bai, Y. , Shang, Z. , Dai, X. , Ji, W. , & Li, T. (2020, Jan). A developmental landscape of 3D‐cultured human pre‐gastrulation embryos. Nature, 577(7791), 537–542. 10.1038/s41586-019-1875-y 31830756

[dgd12715-bib-0088] Yeom, Y. I. , Fuhrmann, G. , Ovitt, C. E. , Brehm, A. , Ohbo, K. , Gross, M. , Hubner, K. , & Scholer, H. R. (1996, Mar). Germline regulatory element of Oct‐4 specific for the totipotent cycle of embryonal cells. Development, 122(3), 881–894. 8631266 10.1242/dev.122.3.881

[dgd12715-bib-0089] Ying, Q. L. , Wray, J. , Nichols, J. , Batlle‐Morera, L. , Doble, B. , Woodgett, J. , Cohen, P. , & Smith, A. (2008, May 22). The ground state of embryonic stem cell self‐renewal. Nature, 453(7194), 519–523. 10.1038/nature06968 18497825 PMC5328678

[dgd12715-bib-0090] Zhang, S. , Chen, T. , Chen, N. , Gao, D. , Shi, B. , Kong, S. , West, R. C. , Yuan, Y. , Zhi, M. , Wei, Q. , Xiang, J. , Mu, H. , Yue, L. , Lei, X. , Wang, X. , Zhong, L. , Liang, H. , Cao, S. , Belmonte, J. C. I. , … Han, J. (2019, Jan 30). Implantation initiation of self‐assembled embryo‐like structures generated using three types of mouse blastocyst‐derived stem cells. Nat Commun, 10(1), 496. 10.1038/s41467-019-08378-9 30700702 PMC6353907

[dgd12715-bib-0091] Zimmerlin, L. , Park, T. S. , Huo, J. S. , Verma, K. , Pather, S. R. , Talbot, C. C. Jr, Agarwal, J. , Steppan, D. , Zhang, Y. W. , Considine, M. , Guo, H. , Zhong, X. , Gutierrez, C. , Cope, L. , Canto‐Soler, M. V. , Friedman, A. D. , Baylin, S. B. , & Zambidis, E. T. (2016, Dec 1). Tankyrase inhibition promotes a stable human naive pluripotent state with improved functionality. Development, 143(23), 4368–4380. 10.1242/dev.138982 27660325 PMC5201042

